# Do texto à cena: dispositivos legais e trajetórias de cuidado na adoção de crianças com deficiência

**DOI:** 10.1590/0102-311XPT198625

**Published:** 2026-08-10

**Authors:** Luciano Silveira Pacheco de Medeiros, Thiago Ferreira Abreu, Luciano Garcia Lourenção, Giovana Calcagno Gomes, Clarice Alves Bonow

**Affiliations:** 1 Universidade Federal de Pelotas, Pelotas, Brasil.; 2 Universidade Federal do Rio Grande, Rio Grande, Brasil.

**Keywords:** Adoção, Crianças com Deficiência, Cuidadores, Atenção Primária à Saúde, Acesso aos Serviços de Saúde, Adoption, Children with Disability, Caregivers, Primary Health Care, Access to Health Services, Adopción, Niños con Discapacidad, Cuidadores, Atención Primaria de Salud, Acceso a los Servicios de Salud

## Abstract

Este estudo teve o objetivo de analisar, pela Análise de Discurso Crítica, como cinco dispositivos legais são ativados, bloqueados ou reconfigurados nas trajetórias de cuidado de famílias adotivas de crianças com deficiência e com quais efeitos em participação, previsibilidade, conforto e continuidade. Trata-se de um estudo qualitativo analítico-descritivo, com 11 cuidadores. Foram realizadas entrevistas presenciais, semiestruturadas, entre 2023 e 2024, transcritas e analisadas pelo modelo tridimensional. Constatou-se que textos normativos entram na cena por práticas e enunciados que ora destravam, ora erodem direitos. Identificaram-se estratégias de bloqueio, *gatekeeping*, burocratização da diferença e neutralização pelo laudo - associadas à queda dos marcadores; e mediações de baixo custo - profissional-ponte, protocolos leves, transparência de filas e ajustes razoáveis pactuados - associadas ao aumento dos marcadores. Variações apareceram por arranjo familiar e âmbito institucional. A retextualização dos dispositivos depende da definição de situação e da negociação de papéis; quando a filiação é publicizada e a coordenação existe, trajetos se encurtam e ansiedades diminuem; quando prevalece a porta giratória baseada em laudo, multiplicam-se remarcações e “recomeços”. Concluiu-se que quem cuida importa, mas como a rede responde importa tanto quanto. Recomenda-se instituir profissional-ponte, coconstruir protocolos leves e ajustes razoáveis, e garantir transparência de filas como infraestrutura mínima para transformar direito previsto em direito vivido; sugere-se ampliar cenários e incluir vozes profissionais em estudos futuros.

## Abstract (en)

This study aimed to analyze, through Critical Discourse Analysis, how five legal devices are activated, blocked, or reconfigured in the care trajectories of adoptive families of children with disabilities and with what effects on participation, predictability, comfort and continuity. This is a qualitative, analytical-descriptive study; with 11 caregivers. Semi-structured face-to-face interviews were conducted between 2023 and 2024, transcribed and analyzed by the three-dimensional model. It was found that normative texts enter the scene through practices and statements that sometimes unlock, sometimes erode rights. Strategies of blocking, gatekeeping, bureaucratization of the difference and neutralization by the report were identified - associated with the drop in markers; and low-cost mediations - bridge professional, light protocols, transparency of queues and reasonable adjustments agreed upon - associated with the increase in markers. Variations appeared by family arrangement and institutional scope. The re-textualization of the provisions depends on the definition of the situation and the negotiation of roles; when affiliation is publicized and coordination exists, paths are shortened and anxieties decrease; when the revolving door based on a report prevails, rescheduling and “new beginnings” multiply. It was concluded that the caregiver matters, but how the network responds matters just as much. It is recommended to institute a bridge professional, co-construct light protocols and reasonable adjustments, and ensure transparency of queues as a minimum infrastructure to transform a right provided for into a right lived; It is suggested to expand scenarios and include professional voices in future studies.

## Resumen

Este estudio tuvo como objetivo analizar, mediante el Análisis Crítico del Discurso, cómo se activan, bloquean o reconfiguran cinco dispositivos legales en las trayectorias de cuidado de familias que adoptaron niños con discapacidad, y qué efectos tienen en la participación, previsibilidad, comodidad y continuidad. Se trata de un estudio cualitativo analítico-descriptivo; realizado con 11 cuidadores. Se realizaron entrevistas presenciales, semiestructuradas, entre 2023 y 2024, que se transcribieron y analizaron mediante el modelo tridimensional. Se constató que los textos normativos entran en escena a través de prácticas y enunciados que, en ocasiones, desbloquean derechos y, en otras, los vulneran. Se identificaron estrategias de bloqueo, *gatekeeping*, burocratización de la diferencia y neutralización mediante informe -asociadas a la disminución de los marcadores-; y mediaciones de bajo costo -profesional-puente, protocolos ligeros, transparencia en las listas de espera y ajustes razonables pactados- asociadas al aumento de los marcadores. Surgieron variaciones según el arreglo familiar y el ámbito institucional. La retextualización de los dispositivos depende de la definición de la situación y la negociación de roles; cuando se hace pública la filiación y existe coordinación, los caminos se acortan y las ansiedades disminuyen; cuando prevalece la puerta giratoria basada en un informe, se multiplican las reprogramaciones y los “nuevos comienzos”. Se concluyó que quien cuida es tan importante como la manera como responde la red. Se recomienda instituir el profesional-puente, coconstruir protocolos ligeros y ajustes razonables, además de garantizar la transparencia en las listas de espera como la infraestructura mínima para convertir el derecho previsto en derecho vivido; se sugiere ampliar los escenarios e incluir voces profesionales en estudios futuros.

## Introdução

No Brasil, a promessa de inclusão inscrita em marcos legais convive com rotinas que, na prática, recolocam nas famílias a tarefa de “fazer a rede existir”. Entre protocolos, laudos, filas e mudanças de regra, o caminho da lei até a cena cotidiana - matrícula escolar, acesso a terapias, transporte, obtenção de benefícios - costuma ser sinuoso ^1^ ^,^ ^2^. Essa distância entre direito previsto e direito vivido tende a ser mais aguda em famílias adotivas de crianças com deficiência, que precisam afirmar a filiação, estabilizar rotinas e negociar com serviços de responsividade variável ^1^ ^,^ ^2^.

Essa distância tende a intensificar quando se trata da adoção de crianças com deficiência, contexto em que a filiação precisa ser continuamente afirmada e reconhecida, ao mesmo tempo em que famílias negociam com dispositivos legais e institucionais historicamente atravessados por práticas capacitistas, burocráticas e seletivas ^1^ ^,^ ^2^. Nesses casos, o cuidado deixa de ser apenas uma resposta técnica às necessidades da criança e passa a envolver disputas simbólicas, administrativas e normativas que moldam as trajetórias familiares no cotidiano.

A adoção de crianças com deficiência constitui uma experiência social e institucional marcada por camadas simultâneas de normatividade. À filiação adotiva - historicamente regulada por dispositivos jurídicos, morais e afetivos - soma-se a deficiência, frequentemente administrada por lógicas biomédicas, educacionais e assistenciais que condicionam o acesso a direitos à apresentação de diagnósticos, laudos e comprovações reiteradas ^3^ ^,^ ^4^. Essa sobreposição produz trajetórias de cuidado singulares, nas quais o reconhecimento da criança como filha e como sujeito de direitos não é automático, mas negociado continuamente nas interações com serviços de saúde, educação e assistência social. Analisar esse contexto permite compreender como dispositivos legais são ativados, bloqueados ou reconfigurados no cotidiano, com efeitos concretos sobre a vida das famílias ^3^ ^,^ ^4^.

Selecionamos cinco dispositivos que, à luz do *corpus*, ritmam o cuidado: (i) Estatuto da Criança e do Adolescente (ECA) ^5^; (ii) Lei Brasileira de Inclusão/Estatuto da Pessoa com Deficiência (LBI) ^6^; (iii) Educação Inclusiva/Atendimento Educacional Especializado (AEE) ^7^; (iv) Rede de Cuidados à Pessoa com Deficiência (RCPD) ^8^ e Atenção Primária à Saúde (APS); e (v) Benefício de Prestação Continuada (BPC) ^9^, articulado aos Centros de Referência de Assistência Social (CRAS) e aos Centros de Referência Especializado de Assistência Social (CREAS). Esses textos normativos atravessam saúde, educação e assistência social e são reiteradamente acionados, retextualizados ou bloqueados nas trajetórias narradas.

A literatura descreve barreiras de acesso, lacunas de cobertura e avaliações de implementação, mas carecemos de descrições processuais do discurso que explicitem como o texto normativo é apropriado, exigido, deslocado ou neutralizado nas interações ^10^ ^,^ ^11^. Interessa-nos, portanto, mapear estratégias discursivas - p.ex.: deslocamento do ônus (“*traga mais um laudo*”), *gatekeeping* (“*não estamos preparados*”), burocratização da diferença (ajustes razoáveis como favor), retextualização afirmativa (quando equipes traduzem a norma em procedimentos simples) e pedagogia institucional (famílias ensinando serviços a operar o dispositivo) - e seus efeitos observáveis no cotidiano ^10^ ^,^ ^11^ ^,^ ^12^.

Operamos com a intertextualidade entre dispositivos legais e cenas narradas pelos participantes, tomando falas *ipsi litteris* como material empírico central. Para observar efeitos sem quantificação, mobilizamos quatro marcadores operacionais: participação (presença e engajamento), previsibilidade (horizontes realistas, menos remarcações e “não atendidos”), conforto (manejo de sensibilidades e queda da ansiedade) e continuidade (vínculos e seguimento sem rupturas). O foco recai menos nos textos legais em si e mais em como são enunciados, exigidos ou flexibilizados nas práticas - e quais mediações discursivas convertem peregrinação em trajetos mínimos viáveis ^13^.

Neste estudo, o cuidado é compreendido como uma prática relacional, situada e intersetorial, produzida na negociação entre famílias e instituições, atravessada por dispositivos normativos que podem tanto viabilizar quanto bloquear trajetórias de participação, previsibilidade, conforto e continuidade no cotidiano.

Pesquisas brasileiras e latino-americanas têm apontado que, no campo da deficiência, o cuidado tende a ser produzido em contextos de descontinuidade institucional, exigindo das famílias estratégias permanentes de mediação com serviços de saúde, educação e assistência social, mesmo em cenários de ampliação normativa de direitos ^14^ ^,^ ^15^ ^,^ ^16^.

Este estudo tem por objetivo analisar, pela Análise de Discurso Crítica (ADC), como cinco dispositivos legais são ativados, bloqueados ou reconfigurados nas trajetórias de cuidado de famílias adotivas de crianças com deficiência e com quais efeitos em participação, previsibilidade, conforto e continuidade. Parte da seguinte questão norteadora: como ECA, LBI, AEE, APS (como porta da RCPD) e BPC são discursivamente ativados, bloqueados ou reconfigurados nas trajetórias de cuidado de famílias adotivas de crianças com deficiência - e com quais efeitos sobre participação, previsibilidade, conforto e continuidade?

## Método

### Desenho do estudo e abordagem analítica

Estudo qualitativo de ADC, na tradição de Fairclough, de caráter analítico-descritivo e exploratório. Adotamos o modelo tridimensional de Fairclough para examinar: (i) o texto (escolhas léxico-gramaticais, modalizações, vozes, nominalizações); (ii) a prática discursiva (como os textos são produzidos, circulam e são retextualizados nas interações família/serviços); e (iii) a prática social (arranjos institucionais, relações de poder e capacitismo que enquadram o cuidado). Nessa moldura, os dispositivos ECA, LBI, AEE, APS/RCPD e BPC/CRAS-CREAS são tratados como intertextos regulatórios que são acionados, condicionados ou neutralizados no cotidiano ^12^.

A opção pela ADC na tradição de Fairclough fundamenta-se na possibilidade de articular texto, interação e estrutura social, permitindo examinar como dispositivos legais são recontextualizados nas práticas cotidianas ^17^. Diferentemente de abordagens discursivas centradas exclusivamente na linguagem ou na experiência subjetiva, a ADC empregada neste estudo enfatiza as relações de poder, os processos de legitimação e os efeitos materiais do discurso, possibilitando compreender como enunciados institucionais produzem consequências concretas nas trajetórias de cuidado das famílias ^17^.

### Cenário do estudo

O estudo foi realizado em um município de porte médio do Sul do Brasil (Rio Grande, Estado do Rio Grande do Sul), tendo como foco o circuito intersetorial por onde circulam famílias adotivas de crianças com deficiência. Esse circuito envolve, de forma articulada, três campos institucionais: saúde, educação e assistência social, com interfaces pontuais com o sistema de justiça da infância e juventude.

Na saúde, a rede municipal é organizada pela APS - unidades básicas de saúde/Estratégia Saúde da Família (ESF) - com papel esperado de porta de entrada, referência e apoio matricial para a RCPD. Os fluxos incluem encaminhamentos para reabilitação (fonoaudiologia, terapia ocupacional, fisioterapia), neuropediatria e outros serviços especializados, com heterogeneidade de oferta entre territórios urbanos e periurbanos. As salas de espera, recepções e canais telefônicos funcionam como pontos críticos de mediação (agendamento, confirmação, reagendamento), onde pequenos acordos operacionais podem reduzir “peregrinações”.

Na educação, o cenário abrange a rede municipal de ensino, com AEE ofertado em salas de recursos e/ou por equipes itinerantes. Observam-se variações entre escolas quanto a processos de matrícula, elaboração de planos pedagógicos individualizados, presença de profissionais de apoio e pactuação de ajustes razoáveis (adaptações de rotina, comunicação com a família, manejo sensorial), o que repercute diretamente na permanência escolar e na previsibilidade do cotidiano.

Na assistência social, o estudo considerou o Sistema Único de Assistência Social (SUAS) - CRAS e CREAS - na interface com o BPC e outros apoios materiais/logísticos (laudos, relatórios, orientação documental). A circulação de papéis (requisições, ofícios, relatórios técnicos) e a clareza de fluxos entre serviços configuram o “trabalho institucional” exigido das famílias para transformar direito previsto em direito vivido.

A entrada e circulação no cenário aconteceram principalmente por locais escolhidos pelos participantes (domicílios, equipamentos públicos, espaços comunitários), preservando o anonimato de pessoas e instituições. Ao longo do campo, os cinco dispositivos analisados - ECA, LBI, AEE, RCPD/APS e BPC - aparecem como textos que organizam a prática, isto é, como referenciais normativos e administrativos que orientam decisões, condicionam passagens e modulam os quatro marcadores operacionais adotados no estudo (participação, previsibilidade, conforto e continuidade).

As trajetórias descritas pelos participantes envolveram predominantemente serviços públicos de saúde, educação e assistência social, com circulação ocasional por serviços privados, especialmente quando as filas, a ausência de profissionais ou a demora na pactuação de ajustes inviabilizavam a continuidade do cuidado. As interações ocorreram em contextos institucionais marcados por fluxos fragmentados, variação de critérios entre serviços e dependência de documentos técnicos (laudos, encaminhamentos, relatórios), elementos que configuraram o pano de fundo discursivo no qual os dispositivos legais analisados foram acionados, negociados ou bloqueados.

### Participantes do estudo

Foram incluídos 11 cuidadores principais de crianças com deficiência, residentes em um município do Sul do Brasil, responsáveis pela coordenação cotidiana do cuidado, isto é, pela organização de rotinas, interlocução com escola/serviços e tomada de decisões terapêuticas. Foram elegíveis cuidadores principais de famílias substitutas de crianças/adolescentes com deficiência congênita ou adquirida, transitória ou permanente, física, intelectual ou múltipla, cuja condição estivesse presente antes da adoção ou fosse reconhecida/diagnosticada durante o estágio de convivência (ainda que, à época da entrevista, a adoção já estivesse consolidada). Excluíram-se famílias ainda em processo de adoção (com estágio de convivência vigente) e famílias em situação de tutela judicial.

Os participantes apresentaram perfis heterogêneos quanto à escolaridade, inserção laboral e condições socioeconômicas, abrangendo famílias em situação de maior vulnerabilidade social e famílias com maior capacidade de mobilização institucional. Os arranjos familiares incluíram casais heterossexuais, casal homoafetivo, mãe solo e família intergeracional, o que permitiu observar variações nas estratégias de coordenação do cuidado. Essa diversidade repercutiu diretamente nas trajetórias narradas, especialmente no acesso a serviços especializados, na possibilidade de complementar cuidados fora da rede pública e na forma como dispositivos como o BPC foram acionados ou não, reforçando a importância de uma leitura contextualizada dos achados.

No que se refere ao BPC, observou-se que 4 dos 11 participantes atendiam aos critérios de elegibilidade, considerando-se os requisitos legais relacionados à renda familiar *per capita* e à comprovação de impedimento de longo prazo. Os demais participantes não se enquadravam nesses critérios, o que repercutiu diretamente nas estratégias de acesso a recursos materiais e na organização do cuidado no cotidiano.

### Coleta e produção dos dados

A produção empírica concentrou-se em entrevistas individuais, presenciais, semiestruturadas, realizadas entre novembro de 2023 e junho de 2024, em locais escolhidos pelos participantes (domicílio, serviço ou espaço comunitário), após apresentação dos objetivos e assinatura do Termo de Consentimento Livre e Esclarecido (TCLE). As entrevistas foram gravadas em áudio e transcritas em formato semiverbatim, com anonimização por signos específicos (personagens da mitologia grega) e supressão/agregação de detalhes potencialmente identificadores.

O roteiro temático integrou 33 perguntas abertas e em profundidade, articuladas a módulos focados nos cinco dispositivos selecionados - ECA, LBI/Estatuto da Pessoa com Deficiência, AEE/Educação Inclusiva, RCPD/APS e BPC/CRAS-CREAS. Em cada módulo, solicitou-se que os cuidadores narrassem episódios concretos, indo da promessa legal à cena cotidiana, descrevendo quem acionou o quê, quais documentos/etapas foram requeridos, onde houve barreiras ou facilidades e quais efeitos perceberam nos quatro marcadores do estudo (participação, previsibilidade, conforto e continuidade).

Para favorecer a memória processual e a explicitação de cadeias intertextuais (no sentido da ACD de Fairclough) ^12^, utilizamos perguntas norteadoras, tais como: “conte o passo a passo”, “o que veio antes e depois?”, “quem falou com quem?” e “o que mudou na rotina?”.

Após cada encontro, o pesquisador elaborou notas de campo e memos analíticos (impressões, hipóteses, estratégias discursivas emergentes, questões para checagem factual), que orientaram os ajustes finos no roteiro e a seleção de cenas nos encontros seguintes (comparação constante).

### Análise e tratamento dos dados

A análise foi manual, ancorada na ADC de Fairclough, operando nas três dimensões: texto (recursos léxico-gramaticais), prática discursiva (produção/circulação/retextualização) e prática social (arranjos institucionais) ^17^ ^,^ ^18^. As transcrições (*ipsis litteris*/semiverbatim) foram organizadas com notas e memos em planilhas com pseudônimo, serviço, dispositivo citado (ECA, LBI, AEE, APS/RCPD, BPC) e tipo de fonte textual (norma pública; “texto em uso” capturado ou referido), constituindo trilha auditável.

Essa etapa seguiu quatro momentos distintos: (i) codificação textual de modalizações, vozes ativa/passiva, nominalizações, apagamento/atribuição de agência, condicionalidades e discurso reportado, registrando quem fala/para quem/em qual posição; (ii) indexação por dispositivo e marcação de intertextos - laudo, encaminhamento, protocolo, PEI (plano educacional individualizado)/PDI (plano de desenvolvimento individual) -, quando disponíveis; (iii) estratégias discursivas - desenvolvimento de *codebook* iterativo para deslocamento do ônus, *gatekeeping*, burocratização da diferença, neutralização pelo laudo, retextualização afirmativa e pedagogia institucional; e, (iv) síntese em blocos “norma-cena” (com fala *ipsi litteris*) e indicação do efeito sobre os marcadores (participação, previsibilidade, conforto, continuidade, ↑/↓), seguidos de matriz transversal (dispositivo × estratégia × mediação × marcador) para comparação constante entre casos/arranjos familiares e busca de casos negativos ^17^ ^,^ ^18^.

O estudo seguiu padrões do reporte qualitativo *Consolidated Criteria for Reporting Qualitative Research* (COREQ-32; Critérios Consolidados para o Relato de Pesquisas Qualitativas), adotando procedimentos para credibilidade, dependabilidade, confirmabilidade e transferibilidade ^19^.

### Aspectos éticos

A pesquisa atendeu às diretrizes da *Resolução nº 510/2016* do Conselho Nacional de Saúde e obteve aprovação do Comitê de Ética em Pesquisa da Faculdade de Enfermagem/Universidade Fedeal de Pelotas (parecer nº 6.532.379; CAAE 75718623.8.000.5316).

Para resguardar a privacidade e o anonimato, os participantes foram identificados por códigos e descritores analíticos (p.ex.: Cuidador 1, pai adotivo), garantindo-se o direito de desistência a qualquer momento, sem qualquer prejuízo. Foi informado que os resultados parciais e finais do estudo seriam compartilhados com os participantes e divulgados em meios técnico-científicos.

Com vistas a garantir a confidencialidade dos participantes, os excertos foram submetidos à edição mínima, restrita à retirada de elementos identificadores e a ajustes de legibilidade, sem alteração de sentido.

## Resultados

Os resultados são apresentados como sequências texto-trabalho-efeito, mostrando como dispositivos legais e organizacionais atravessam o cotidiano e se materializam em quatro marcadores do cuidado (participação, previsibilidade, conforto e continuidade).

A leitura avança do caso à síntese, com trechos de entrevistas que exemplificam arranjos e mediações em cinco áreas: ECA - convivência, filiação e vínculos de irmãos; LBI - acessibilidade e ajustes razoáveis; AEE - matrícula, permanência e mediações pedagógicas; APS/RCPD - coordenação, referência e tempos de espera; e BPC/CRAS-CREAS - renda, documentação e circulação de direitos.

### ECA: convivência, filiação e vínculos de irmãos

O ECA aparece como linguagem de legitimidade para a filiação e a preservação de vínculos fraternos, funcionando como referência pública para decisões de guarda, matrícula e garantia de convivência. A decisão de manter irmãos juntos foi tematizada como virada ética que orienta rotas e pactos com instituições, reduz ambiguidades e ancora negociações intersetoriais:

“*Quando fomos apresentados ao perfil dos três irmãos algo se encaixou. Mesmo diante dos desafios, das deficiências, do racismo estrutural que sabíamos que viriam junto, não houve mais hesitação. Estávamos prontos. E sabíamos disso. A decisão não foi um gesto heroico, muito menos um movimento inconsequente. Foi um gesto pensado, profundo, sustentado por amor, estrutura e, sobretudo, compromisso com a vida desses três pequenos que, desde então, são nossos filhos - tão filhos quanto nossos filhos biológicos*” (Cuidador 1, pai adotivo).

Na fala desse participante, o ECA opera como intertexto de legitimação da filiação, ainda que não seja explicitamente citado. A ênfase na decisão “*pensada*” e no compromisso com a vida desloca a adoção do campo da excepcionalidade moral para o campo do direito, produzindo um efeito discursivo de estabilização da filiação. Ao afirmar que os filhos adotivos são “*tão filhos quanto*” os biológicos, o participante antecipa e neutraliza possíveis questionamentos institucionais sobre pertencimento, o que tende a elevar os marcadores de participação e previsibilidade nas interações subsequentes com serviços e escolas.

“*A relação entre os nossos filhos é, pra mim, uma das coisas mais bonitas e, ao mesmo tempo, mais difíceis de descrever. Porque ela escapa das formas tradicionais que a gente costuma imaginar quando pensa em irmãos. Eles não brincam de luta no sofá. Não disputam o controle remoto da TV. Não dividem segredos no quarto. Mas compartilham uma forma de presença que é profunda, silenciosa, e, sobretudo, construída com muito afeto. O mais velho cresceu sabendo que o irmão precisava de cuidados diferentes*” (Cuidador 2, pai adotivo).

Aqui, a filiação é construída discursivamente no cotidiano, por meio da descrição de formas não normativas de vínculo fraterno. O discurso desloca expectativas sociais padronizadas sobre irmandade e introduz uma definição situada de convivência, alinhada ao princípio do melhor interesse da criança previsto no ECA. Esse movimento discursivo funciona como mediação simbólica que sustenta o conforto emocional e a continuidade do cuidado, mesmo na ausência de práticas institucionais plenamente ajustadas.

O reconhecimento da filiação também é narrado como prática encarnada, vivida no cotidiano - gestos, presenças e responsabilidades - que ultrapassa a centralidade documental, produz reconhecimento público da família e frequentemente antecede o ato formal:

“*Sempre houve respeito - nunca houve hostilidade explícita - mas a distância emocional entre as duas gerava tensão dentro da casa, o que impactava, inevitavelmente, o ambiente familiar como um todo. Houve também, especialmente da minha parte, um processo interno de ajuste de expectativas. Como mãe, mesmo sendo médica e conhecendo teoricamente o funcionamento do espectro autista, levei tempo para entender que o afeto da minha filha não se manifestaria da forma como eu esperava. Foi frustrante...*” (Cuidadora 3, mãe adotiva).

“*Havia uma diferença no modo como ele percebia o mundo e interagia com ele. Só que, naquele momento, nós ainda não associávamos isso diretamente ao espectro autista. Algumas pessoas até chegaram a levantar essa hipótese e começamos a ler um pouco sobre o assunto, mas não mergulhamos profundamente em pesquisas ou artigos - não fomos ao Google devorar informações. Em vez disso, buscamos ajuda profissional. Ao longo de dois anos - entre 2020, quando ele chegou, e 2022, quando o diagnóstico foi fechado - percorremos um caminho de consultas, avaliações...*” (Cuidadora 4, mãe adotiva).

Nos relatos, observa-se que o reconhecimento da filiação não é apenas jurídico, mas processual e relacional. A tensão entre expectativas parentais e formas singulares de expressão afetiva evidencia como a filiação adotiva de crianças com deficiência exige renegociações contínuas de sentido. Quando esse processo não é reconhecido institucionalmente, a família assume sozinha o trabalho de adaptação, com impactos diretos sobre o marcador conforto. Por outro lado, quando a filiação é publicizada e reconhecida nos serviços, reduz-se a necessidade de comprovação reiterada, fortalecendo a previsibilidade das rotinas.

As narrativas dos participantes mostram que o ECA atua menos como norma abstrata e mais como recurso discursivo mobilizado pelas famílias para sustentar a filiação no cotidiano. Quando esse reconhecimento é incorporado pelas instituições, observam-se trajetórias mais curtas, menor ansiedade e maior continuidade do cuidado. Quando não o é, a filiação precisa ser reiteradamente performada, deslocando para as famílias o ônus de estabilizar aquilo que já é direito previsto.

### LBI: acessibilidade e ajustes razoáveis

A LBI emerge quando famílias demandam adaptações razoáveis - ajustes curriculares, apoio do AEE, manejo sensorial e acessibilidade comunicacional - e contestam a “laudificação” de direitos, isto é, a conversão de garantias legais em exigência contínua de laudos. No ambiente escolar, a exigência prévia de diagnósticos formais, somada à morosidade para pactuar e implementar ajustes, reduz a presença e o engajamento, intensifica faltas/remarcações e compromete a previsibilidade das rotinas:

“*O direito ao AEE está na lei, tá escrito, é bonito no papel. Mas fazer isso acontecer na prática* (...) *é outra história. Primeiro, a escola não sabia direito se ele tinha direito ou não. Depois, não tinha professor formado. Depois, tinha o professor, mas não tinha espaço. Foi um vai e vem. Mas a gente insistiu. Fomos na Secretaria de Educação. Levamos laudo. Levamos relatório. Levamos paciência. E, depois de um tempo, conseguimos. Ele começou a frequentar o AEE duas vezes por semana. Foi um avanço. Pequeno, mas importante*” (Cuidador 5, pai adotivo).

“*Começou com a neuropediatra. Ali, de cara, já bateu o primeiro muro: ‘não tem vaga’, ‘precisa de diagnóstico formal para o encaminhamento’, ‘a fila é longa’, ‘não temos neurologista na rede nesse momento’. Tive que brigar, insistir, juntar laudos de fonoaudióloga e da psicóloga escolar para conseguir uma avaliação com a neuropediatra pelo SUS. Isso levou meses. Enquanto isso, busquei uma profissional particular, pagando por fora - e com sacrifício - só para que ele não ficasse sem acompanhamento*” (Cuidadora 6, mãe adotiva).

### AEE: matrícula, permanência e mediações pedagógicas

O AEE e as equipes de inclusão funcionam como pontos de inflexão: quando existem pactos simples e explícitos (horário flexível, comunicação ágil, objetivos claros em PEI/planos de apoio), ampliam-se a participação e o conforto e as rotinas ficam mais previsíveis; na ausência desses acordos, proliferam “idas e vindas”, pedidos de novo laudo, remarcações e perdas de atendimento, com impacto direto na continuidade:

“*Tivemos experiências muito difíceis no início, especialmente com a primeira escola da rede pública que ele frequentou, onde não havia preparo adequado para acolher uma criança com autismo. Essa vivência acabou marcando profundamente o meu filho, a ponto de ele não conseguir nem passar em frente à escola sem manifestar angústia. Foi doloroso perceber o quanto a ausência de práticas inclusivas pode não apenas dificultar a aprendizagem, mas também causar sofrimento emocional real. Depois dessa experiência, optamos por uma escola da rede privada*” (Cuidadora 4, mãe adotiva).

### APS/RCPD: coordenação, referência e tempos de espera

Na saúde, a experiência de filas prolongadas e opacidade de fluxos - critérios incertos, ausência de previsão e comunicação fragmentada - convive com efeitos positivos quando há referência e apoio matricial: a presença de um profissional-ponte, com planos compartilhados e devolutivas entre APS e especializada, organiza prioridades clínicas, encurta trajetos, reduz “peregrinações” e remarcações, e aumenta a previsibilidade.

Nesses arranjos, pactos de retorno já agendado, canais diretos para dúvidas e ajustes razoáveis sustentam a continuidade do cuidado e diminuem a ansiedade familiar, transformando demandas dispersas em itinerários mínimos viáveis:

“*A médica da UBS foi muito humana. Fez o acolhimento inicial, pediu exames, nos escutou com paciência. Mas ali já percebemos que, embora houvesse boa vontade, a estrutura era muito limitada. Os encaminhamentos demoravam, as especialidades tinham fila. O primeiro pedido de neurologista, por exemplo, demoraria meses. A fonoaudióloga atendia só uma vez por semana. A TO tinha lista de espera. Diante disso, e com o agravante de que meu marido estava em casa em tempo integral com ela, nós decidimos buscar complementar esse cuidado fora da rede pública*” (Cuidadora 7, mãe adotiva).

A fala evidencia a dissociação entre acolhimento relacional e capacidade organizacional da APS. Discursivamente, a “boa vontade” aparece como valor moral individual, enquanto a agência institucional é diluída por nominalizações (“*os encaminhamentos demoravam*”, “*tinha fila*”), apagando responsáveis e critérios. Esse movimento desloca o tempo institucional para o tempo da família, reduz a previsibilidade e fragiliza a continuidade, ainda que a participação inicial seja garantida. Na ausência de coordenação explícita, a APS deixa de operar como ordenadora da RCPD e passa a funcionar como ponto de passagem.

“*O SUS dá o básico que ele precisa. Demora, atrasa, mas uma hora sai. O leite especial dele vem por mês, às vezes atrasa, mas chega. O benefício dele ajuda a comprar fralda, as medicações mais caras. E quando precisa de exame mais complicado, eles acabam arranjando. Não é fácil. Não é rápido. Mas é o que temos. E é o que segura a gente. Antes, a gente ia no posto a cada três meses. Agora é toda semana. Tem dias que a gente sai cedo de casa, debaixo de chuva, para pegar lugar. E mesmo com o número na mão, espera horas. Mas a gente vai. Porque precisa*” (Cuidador 8, pai adotivo).

Aqui, a repetição da espera e a naturalização do atraso constroem um discurso de resignação ativa, no qual a família assume a regularidade da presença como estratégia de sobrevivência institucional. A APS é retextualizada como espaço de persistência, não de coordenação, exigindo comparecimentos reiterados para manter o cuidado mínimo. Embora a continuidade seja parcialmente preservada pelo esforço familiar, a previsibilidade permanece baixa e o conforto é comprometido pela exposição constante a filas, intempéries e incertezas.

Nos diferentes relatos, observa-se que a ausência de pactos simples - retorno já agendado, canal direto de comunicação, clareza de critérios e posição na fila - intensifica a “peregrinação” entre os serviços. Discursivamente, a falta de referência se manifesta por condicionalidades vagas (“*aguarde*”, “*quando surgir vaga*”) e por uma lógica de porta giratória baseada em laudos, o que rebaixa os marcadores de previsibilidade e continuidade, e transfere às famílias o trabalho de navegação da rede.

Os achados indicam que a APS e a RCPD operam como dispositivos decisivos para estabilizar ou desorganizar as trajetórias de cuidado. Quando há coordenação explícita - profissional de referência, devolutivas entre níveis de atenção, pactuação de retornos e transparência de fluxos - os trajetos encurtam, a ansiedade diminui e os marcadores de participação, previsibilidade, conforto e continuidade aumentam. Na ausência dessas mediações, a rede se torna opaca e fragmentada, convertendo direito previsto em esforço contínuo das famílias para manter o cuidado em funcionamento.

### BPC/CRAS-CREAS: renda, documentação e circulação de direitos

O BPC e os CRAS/CREAS aparecem como sustentação material - garantindo renda mínima e viabilizando transporte/insumos -, mas cercados por exigências documentais graduais, prazos pouco claros e orientações divergentes entre órgãos, produzindo um vai-e-vem burocrático que compromete a participação e a previsibilidade nas rotinas de cuidado:

“*Do ponto de vista social, o único benefício que solicitamos foi o BPC, e nesse caso até que foi tranquilo. Fizemos o pedido com base nos laudos e relatórios atualizados. Fomos ao CRAS, depois ao INSS. Tivemos que reunir uma série de documentos - comprovante de renda, de residência, número do NIS etc. Mas o processo correu bem. Em poucos meses o benefício foi concedido. E isso ajudou muito. Porque mesmo que a gente não estivesse em vulnerabilidade extrema, o BPC traz um respiro*” (Cuidador 5, pai adotivo).

“*Fui até o CRAS do meu bairro e dei entrada no BPC. Foi um processo longo, desgastante. Precisei apresentar laudos atualizados, comprovação de renda, atestados escolares e de saúde, além de entrevistas socioeconômicas com assistente social. A cada passo, um novo entrave: faltava um carimbo, um formulário estava desatualizado, o cadastro precisava ser renovado. Foram meses até conseguir o deferimento. O BPC não é um favor. É um direito. Mas para acessá-lo, precisei provar o tempo todo que meu filho merecia o que já era dele por lei*” (Cuidadora 6, mãe adotiva).

Em todas as áreas, mediações organizacionais simples (profissional-referência/matriciamento, protocolos leves, transparência de filas e ajustes razoáveis) funcionam como “gatilhos de estabilidade”, elevando os quatro marcadores. Onde faltam, observam-se peregrinações administrativas, maior ansiedade e risco de quebras de continuidade. A articulação entre disposições familiares e responsividade institucional explica variações intercasos, inclusive entre arranjos semelhantes.

## Discussão

Os achados mostram que os dispositivos normativos não operam como pano de fundo neutro; eles entram na cena por meio de práticas e enunciados que podem destravar ou erodir direitos.

A leitura pela ADC (Fairclough) evidenciou um circuito no qual textos regulatórios (ECA, LBI, AEE, APS/RCPD, BPC) ^5^ ^,^ ^6^ ^,^ ^7^ ^,^ ^8^ ^,^ ^9^ são retextualizados nas interações família/serviços, produzindo efeitos observáveis nos quatro marcadores - participação, previsibilidade, conforto e continuidade ^17^ ^,^ ^18^. O padrão é não linear: os mesmos dispositivos que habilitam a inclusão também podem, por certas estratégias discursivas, reinstalar barreiras ^17^ ^,^ ^18^.

Uma primeira contribuição diz respeito às estratégias que bloqueiam. Identificamos deslocamento do ônus (“*traga mais um laudo*”), *gatekeeping* (“*não estamos preparados*”), neutralização pelo laudo (direito convertido em favor condicionado a papel) e burocratização da diferença (ajustes razoáveis tratados como exceção). Tais estratégias baixam a participação (menos presença em escola/terapias), corroem a previsibilidade (remarcações, “*não atendidos*”), elevam a ansiedade (conforto↓) e quebram o seguimento (continuidade↓) ^10^ ^,^ ^11^ ^,^ ^20^.

Em termos de prática discursiva, há apagamentos de agência (voz passiva, indeterminação de responsáveis) e condicionalidades. Tais enfrentamentos deslocam o tempo institucional para o tempo da família, forçando “trabalho de prova” contínuo ^10^ ^,^ ^11^.

Em contrapartida, os achados também destacam estratégias que destravam: retextualização afirmativa (tradução da norma em passo-a-passo simples), pedagogia institucional (quando a família ensina o serviço e o serviço aprende) ^21^ ^,^ ^22^ e mediações organizacionais de baixo custo - profissional-ponte (enfermeira(o) de referência), protocolos leves (roteiros curtos, contatos diretos), transparência de filas e ajustes razoáveis pactuados. Quando presentes, tais mediações elevam os quatro marcadores (participação↑, previsibilidade↑, conforto↑, continuidade↑) ao reduzir ruídos comunicacionais e encurtar trajetos ^10^ ^,^ ^23^.

As variações por arranjo familiar reforçam que quem cuida importa, mas como a instituição responde importa tanto quanto. Nos casais heterossexuais, a conjugalidade funciona como amortecedor organizacional (revezamento de tarefas), mas não substitui mediações: sem protocolo leve e transparência, a previsibilidade segue frágil ^24^ ^,^ ^25^ ^,^ ^26^ ^,^ ^27^. No casal homoafetivo, a publicização da filiação opera como pedagogia institucional, gerando pactos operacionais (assinaturas, prontuários, retirada na escola) que beneficiam outros casos. No caso da mãe solo, concentrar sozinha toda a coordenação tem um custo alto: ela precisa comprovar tudo o tempo todo. Encaminhamentos feitos com alguém do serviço apresentando o caso e já ajudando a marcar o próximo passo, junto de uma pessoa de referência que acompanha a família, reduzem esse peso e ajudam a manter as rotinas funcionando ^27^ ^,^ ^28^ ^,^ ^29^.

Na família intergeracional, logística e saúde do cuidador modulam a aderência; por isso, APS organizada (referência/matriciamento) e AEE consistente tornam-se pontos de ancoragem para a continuidade ^23^ ^,^ ^30^ ^,^ ^31^ ^,^ ^32^ ^,^ ^33^. O recorte adoção + deficiência adiciona uma camada específica: a performação pública da filiação (especialmente na escola e nos serviços) atua como reconhecimento e reduz ambivalências institucionais.

O ECA, quando retextualizado de modo afirmativo, desloca dúvidas sobre o “lugar de fala” da família e abre canais operacionais (manter irmãos juntos, garantir permanência, reduzir prova redundante). Já a LBI/AEE, quando lidas como “exceção” e não como padrão de atendimento, tendem a produzir laudificação e porta giratória ^34^ ^,^ ^35^ ^,^ ^36^.

## Considerações finais

Este estudo evidenciou que o desfecho das trajetórias de cuidado na adoção de crianças com deficiência depende menos de “boa vontade” individual e mais de como os dispositivos normativos são enunciados e operados nas interações cotidianas. Com base na ADC (Fairclough), mostramos que certas estratégias discursivas - deslocamento do ônus, *gatekeeping*, burocratização da diferença e neutralização pelo laudo - corroem a participação, a previsibilidade, o conforto e a continuidade, ao passo que mediações simples e consistentes - profissional-ponte, protocolos leves, transparência de filas e ajustes razoáveis pactuados - convertem direito previsto em direito vivido.

Do ponto de vista prático e gerencial, os achados apontam alvos operacionais claros para Enfermagem, APS/RCPD e educação inclusiva: (i) instituir profissional-ponte com responsabilidade explícita de navegação e devolutiva entre pontos; (ii) adotar protocolos leves (uma página), com contatos diretos, critérios simples e procedimento de retorno; (iii) pactuar ajustes razoáveis (janelas de atendimento, manejo sensorial, flexibilização de horários) como rotina, não exceção; (iv) garantir transparência de filas (posição, previsão, canal para dúvidas); e (v) produzir documentos persuasivos sintéticos que acompanhem a criança entre os serviços.

Medidas de baixo custo e alto impacto que dialogam diretamente com os quatro marcadores e oferecem uma linguagem comum para monitorar efeitos no tempo. Reconhecemos limites contextuais (*corpus* municipal).

Como próximos passos, sugerimos ampliar cenários (multimunicípio), coletar sistematicamente documentos institucionais e incluir vozes de profissionais (enfermagem, gestão escolar, assistência) para cotejar estratégias dos dois lados da interação. Ainda assim, a convergência intercasos e a coerência excerto↔efeito sustentam a transferibilidade dos achados para as redes que enfrentam problemas semelhantes de coordenação e reconhecimento.

Quem cuida importa, e como a rede responde importa tanto quanto. Coordenação concreta, comunicação clara e ajustes razoáveis compõem a infraestrutura mínima para que a adoção de crianças com deficiência se traduza em vida cotidiana vivível, com participação, previsibilidade, conforto e continuidade.

## Data Availability

Os dados de pesquisa estão disponíveis mediante solicitação ao autor de correspondência.
